# Low Potassium Dialysate as a Protective Factor of Sudden Cardiac Death in Hemodialysis Patients with Hyperkalemia

**DOI:** 10.1371/journal.pone.0139886

**Published:** 2015-10-06

**Authors:** Chien-Wei Huang, Min-Jing Lee, Po-Tsang Lee, Chih-Yang Hsu, Wei-Chieh Huang, Chien-Liang Chen, Kang-Ju Chou, Hua-Chang Fang

**Affiliations:** 1 Division of Nephrology, Department of Internal Medicine, Kaohsiung Veterans General Hospital, Kaohsiung, Taiwan; 2 Department of Child and Adolescent Psychiatry, Kaohsiung Chang Gung Memorial Hospital and Chang Gung University College of Medicine, Kaohsiung, Taiwan; 3 National Yang-Ming University, School of Medicine, Taipei, Taiwan; The University of Tokyo, JAPAN

## Abstract

**Aim:**

Hyperkalemia increases the risk of sudden cardiac death (SCD) in hemodialysis patients. Our objective was to determine the association between administering low potassium dialysate to hyperkalemic hemodialysis patients and SCD.

**Methods:**

We conducted a retrospective cohort study with patients undergoing maintenance hemodialysis from May 1, 2006, through December 31, 2013. The dialysate composition was adjusted over time according to monthly laboratory results. A 1.0 mEq/L potassium dialysate was applied in patients with predialysis hyperkalemia (>5.5 mEq/L) and was included as a time-dependent confounding factor. The clinical characteristics of enrolled patients, the incidence and timing of SCD and risk factors for all-cause mortality and SCD were analyzed.

**Results:**

There were 312 patients on maintenance hemodialysis during the study period. One hundred and fifty-seven patients had been dialyzed against a 1.0 mEq/L potassium dialysate at least once. The rates of all-cause mortality and SCD were 48.17 and 20.74 per 1000 patient-years, respectively. A 1.12-fold increase in the risk of SCD in the 24-hour period starting with the hemodialysis procedure and a 1.36-fold increase in the 24 hours preceding a weekly cycle were found (*p* = 0.017). Multivariate Cox proportional hazards models showed that age, diabetes mellitus and predialysis hyperkalemia (>5.0 mEq/L) were significant predictors of all-cause mortality and SCD. Exposure to 1.0 mEq/L potassium dialysate, Kt/V, and serum albumin were independent protective factors against all-cause mortality. Only exposure to 1.0 mEq/L potassium dialysate significantly prevented SCD (hazard ratio = 0.33, 95% CI = 0.13–0.85).

**Conclusions:**

Using low potassium dialysate in hyperkalemic hemodialysis patients may prevent SCD.

## Introduction

Sudden cardiac death (SCD) is the leading cause of death in hemodialysis patients. In the United States Renal Data System (USRDS) database, 26.9% of all-cause mortalities in prevalent dialysis patients between 2009 and 2011 were attributed to cardiac arrest or arrhythmias. The incidence of SCD in hemodialysis patients was 49.2 per 1000 patient-years in 2011, which is much higher than that of the general population [1–3]. Hence, prevention of SCD in hemodialysis patients is of paramount importance.

Several risk factors for SCD in hemodialysis patients have been proposed, including traditional cardiac risk factors and dialysis-specific risk factors [3–9]. Among these risk factors, hyperkalemia is a well-known and significant risk factor for overall mortality and SCD in hemodialysis patients. It is primarily caused by reduced renal potassium excretion in patients with end-stage renal disease (ESRD). In daily practice, dialysis adequacy is optimized, patients are advised to avoid potassium-rich food, and sometimes potassium binding resins are used to increase gastrointestinal excretion of potassium. Furthermore, most patients receive hemodialysis with relatively low potassium dialysate (usually 2.0 mEq/L) to maintain the serum potassium concentration within a desirable range. Kovesdy et al. found that hyperkalemic patients with predialysis serum potassium concentrations ≥5.0 mEq/L have longer survival times when they undergo hemodialysis against a low potassium dialysate [10]. In contrast, the use of low potassium dialysate may lead to adverse electrocardiographic changes during and after hemodialysis [11–16]. Several observational studies have indicated that hemodialysis against a low potassium dialysate might increase the risk of SCD, mainly in patients without predialysis hyperkalemia [17–19]. The use of low potassium dialysate in hemodialysis patients with predialysis hyperkalemia, therefore, remains controversial. In this study, we tested the hypothesis that the use of low potassium dialysate (1.0 mEq/L) in hemodialysis patients with predialysis hyperkalemia reduces the risk of SCD.

## Materials and Methods

### Study Design and Participants

**Fig 1:**
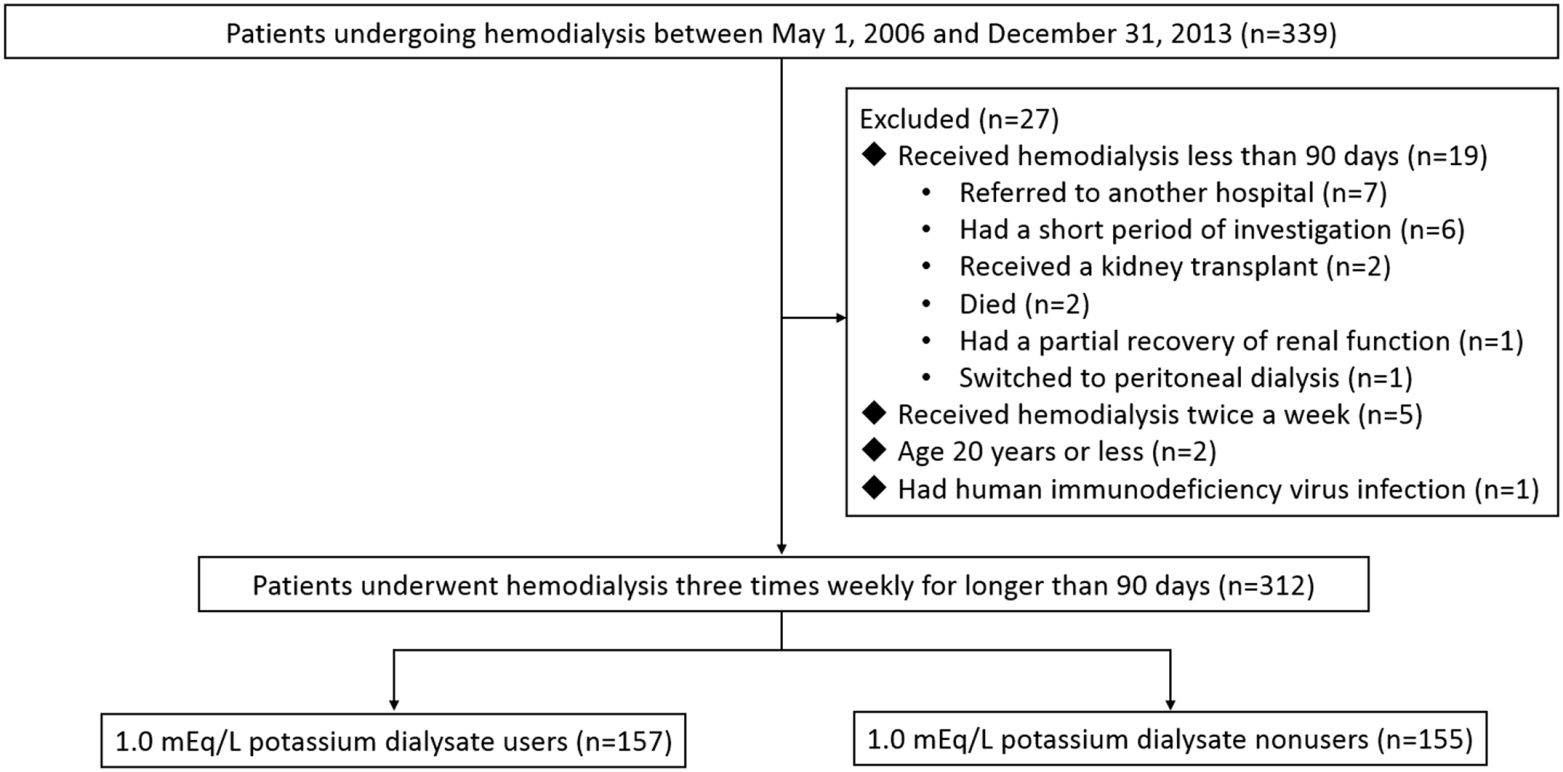
Flowchart of the patient selection process. Patients who had been exposed to a 1.0 mEq/L potassium dialysate at least once were defined as 1.0 mEq/L potassium dialysate users.

The study was designed as a retrospective cohort study. We enrolled patients with ESRD on maintenance hemodialysis, defined as regular hemodialysis three times weekly for longer than 90 days, from May 1, 2006, through December 31, 2013. We excluded patients younger than 20 years and those who had received hemodialysis twice a week. Patients who died, received a kidney transplant, switched to peritoneal dialysis, were referred to another hospital, or had sufficient improvement in renal function that they could be taken off hemodialysis, between the time of recruitment and the 90^th^ day of the investigation, were also excluded. In addition, we excluded a patient with human immunodeficiency virus infection. Ultimately, 312 patients in the same hemodialysis unit of one medical center were selected for the study (Fig 1). Demographic data and underlying comorbid conditions, including coronary artery disease, diabetes mellitus, ejection fraction, electrocardiographic findings, cardiothoracic ratio, cause of ESRD, and type of vascular access, were obtained by chart review. Ejection fraction was measured by transthoracic echocardiogram. The findings of the electrocardiogram included atrial fibrillation, conduction block, atrioventricular block, and corrected QT (QTc) prolongation. The QTc prolongation was defined as a QTc interval >0.45 sec in men and >0.47 sec in women. The cardiothoracic ratio was calculated by dividing the widest transverse diameter of the cardiac silhouette by the widest transverse diameter of the thorax above the diaphragm. We defined dialysis vintage as the duration of time between the first day of hemodialysis and the first day that the patient entered the cohort.

### Ethics Statement

This retrospective cohort study was conducted without involving patient intervention or obtaining clinical specimens. The personal information had been de-identified and all the data were analyzed anonymously. Hence, the informed consent for this study was waived. This study and the waiving of informed consent for this study were approved by the Institutional Review Board of Kaohsiung Veterans General Hospital (VGHKS15-CT2-07).

### Hemodialysis Prescription

All enrolled patients had been on maintenance hemodialysis three times weekly. The duration of each hemodialysis session varied from 3.5 to 4 hours, with more than 95% of the patients undergoing 4-hour sessions. Artificial polysulfone kidneys with a surface area of 1.4, 1.6, 1.8, or 2.1 m^2^ were prescribed according to the body size of the patients and monthly urea kinetic modeling measurements (Kt/V of urea). Dialysis adequacy is optimized to achieve a target Kt/V of 1.2 or greater. The dialysate was composed of sodium, 140 mEq/L; calcium, 2.5, 3, or 3.5 mEq/L; potassium, 1.0, 2.0, 2.5 or 3 mEq/L; bicarbonate, 35 mEq/L; and dextrose, 200 mg/dL. The hemodialysis blood flow rates were 250–350 ml/min and dialysate flow rates were 500–700 ml/min. The dialysate temperature was maintained at 36–36.5°C.

Throughout the study period, dietary potassium and phosphate were restricted, sodium polystyrene sulfonate and phosphate binders were used, and dialysate potassium and calcium concentrations were adjusted in order to control serum potassium concentration and optimize the total body calcium and phosphate load. Diuretics were not prescribed in all patients undergoing maintenance hemodialysis. Most of the patients were dialyzed against a dialysate with a potassium concentration of 2.0 mEq/L and calcium concentration of 3.0 mEq/L. Hyperkalemic patients with predialysis serum potassium concentration >5.5 mEq/L were dialyzed against a 1.0 mEq/L potassium dialysate. We switched back to a 2.0 mEq/L potassium dialysate when the predialysis serum potassium concentration declined to a level of 5.0 mEq/L or less. Sodium polystyrene sulfonate, 15 gram orally once daily, was used to treat the patients who persistently had predialysis hyperkalemia even after dialysis against a 1.0 mEq/L potassium dialysate. Patients with a serum calcium concentration >11.0 mg/dL were dialyzed against a 2.5 mEq/L calcium dialysate. We switched back to a 3.0 mEq/L calcium dialysate if patients suffered from intradialytic hypotension or if the predialysis serum calcium concentration declined to a level of 10.5 mg/dL or less.

### Exposure Assessment

Patients who had been dialyzed against a 1.0 mEq/L potassium dialysate at least once were defined as 1.0 mEq/L potassium dialysate users; the remaining patients were defined as 1.0 mEq/L potassium dialysate nonusers. We adjusted the dialysate composition according to the individuals’ characteristics and the monthly laboratory results. The dialysate potassium and calcium concentrations for each hemodialysis patient could vary over time. Hence, we included exposure to 1.0 mEq/L potassium dialysate and to 2.5 mEq/L calcium dialysate as time-dependent confounding factors.

### Laboratory Data

Most blood samples were collected predialysis with the exception of post-dialysis serum urea nitrogen to calculate Kt/V of urea. Monthly laboratory values, including serum levels of urea nitrogen, albumin, hemoglobin, potassium, creatinine, phosphate, and alkaline phosphatase, were measured by automated and standardized methods in a central laboratory of the medical center. Serum concentrations of albumin were measured by bromocresol green assay. Single-pool Kt/V was calculated according to methods described by Daugirdas et al. [20]. These laboratory values were collected to assess the impact of long-term effects in terms of time-averaged variables.

### Outcome measures

We evaluated the effect of the dialysate potassium concentration on serum potassium level. The predialysis serum potassium concentration of patients during hemodialysis with 1.0 mEq/L potassium dialysate was compared to that of patients during hemodialysis with 2.0 mEq/L potassium dialysate. The incidence of hypokalemia, defined as a predialysis serum potassium concentration <4.0 mEq/L, during these two intervals was measured in terms of 100 patient-months. Furthermore, we assessed the influence of using 1.0 mEq/L potassium dialysate on serum potassium level. The predialysis serum potassium concentrations of patients before and one month after hemodialysis with 1.0 mEq/L potassium dialysate were investigated. The incidence of hypokalemia when switching from 2.0 mEq/L to 1.0 mEq/L potassium dialysate was calculated. The drop of serum potassium concentration by 1.0 mEq/L and 2.0 mEq/L potassium dialysate in 1.0 potassium dialysate users was collected. The drop of serum potassium concentration was assessed by the difference of monthly predialysis serum potassium concentrations.

The primary outcomes were all-cause mortality and SCD. SCD was defined as a witnessed unexpected death due to cardiac arrest, cardiac arrhythmia, and/or hyperkalemia, or an unwitnessed, unexpected cardiac arrest or cardiac arrhythmia without an obvious non-cardiac cause. The latter condition occurred during the interval between hemodialysis sessions. The rates of all-cause mortality and SCD were calculated in terms of 1000 patient-years. We recorded the duration of time from the start of hemodialysis to the time of SCD and defined this as the timing of SCD. We divided the timing of SCD into three time periods: 0–24 hour, 24–48 hour and 48–72 hour intervals. We calculated the relative risk as the proportion of observed SCD events in each of the three time intervals versus the expected SCD events, assuming that the SCD events would be evenly distributed over time. The 48–72 hour interval, in other words, the 24 hours prior to the first time of hemodialysis in a week, occurred only once during the week, while the other two time intervals occurred three times weekly. Therefore, we expected one-seventh of the total SCD events to occur in the 48–72 hour interval. The observation period started from the enrollment date to the date of death, to the date when patients’ data were censored as a result of receiving kidney transplant, switching to peritoneal dialysis, referral to other hospital, or coming off hemodialysis because of sufficient renal function improvement, or to December 31, 2013; whichever came first.

### Statistics

All data are expressed as mean±SD, median (interquartile range), or number (percentage), as appropriate. Baseline characteristics and laboratory data were compared between the 1.0 mEq/L potassium dialysate users and nonusers using chi-square tests with or without Yates’ correction for categorical variables, independent t-tests for normally distributed continuous variables, and Mann-Whitney U-tests for skewed continuous variables. The paired t-tests was used to assess the effect of 1.0 mEq/L potassium dialysate on predialysis potassium concentration and the difference of the drop of serum potassium level by 1.0 mEq/L and 2.0 mEq/L potassium dialysate in 1.0 mEq/L dialysate users. We evaluated the significance of the ratio of observed to expected sudden cardiac death in three time periods using chi-square tests. Univariate and multivariate Cox proportional hazards models were used to assess the influence of confounders on primary outcomes. Potential confounders, including age, sex, dialysis vintage, body mass index, exposure to 1.0 mEq/L potassium or 2.5 mEq/L calcium dialysate, coronary artery disease, diabetes mellitus, Kt/V, serum levels of albumin, hemoglobin, potassium, creatinine, phosphate and alkaline phosphatase, were adjusted in the multivariable models. Exposure to 1.0 mEq/L potassium dialysate and to 2.5 mEq/L calcium dialysate were investigated as time-varying covariates, as described in the Exposure Assessment section. Additionally, we analyzed the association between the drop of serum potassium concentration by 1.0 mEq/L or 2.0 mEq/L potassium dialysate and the primary outcome in 1.0 mEq/L potassium dialysate users. The effects of the drop of serum potassium level by 1.0 mEq/L or 2.0 mEq/L potassium dialysate were further adjusted for the same confounding factors in the multivariate Cox proportional hazards model. All *p* values were two-sided, and the significance level was set at 0.05. Statistical analyses were performed using SPSS version 22.0.0 (SPSS Inc., Chicago, IL).

## Results

### Patient characteristics

We included 312 patients who had been on maintenance hemodialysis three times weekly for longer than 90 days in the present study (Fig 1). The mean age, median dialysis vintage and median body mass index of this population were 60 years, 24.6 months and 22 Kg/m^2^, respectively. Of the participants, 52.2% were woman, 21.2% had at least one prescription of a 2.5 mEq/L calcium dialysate, 12.5% had coronary artery disease, and 33.7% had diabetes mellitus (Table 1). 80% of the participants received transthoracic echocardiogram and 75.1% had preserved ejection fraction. As to the findings of the electrocardiogram, 5.1% of the participants had atrial fibrillation, 6.4% had conduction block, 6.1% had atrioventricular block, and 35.9% had QTc prolongation. The median cardiothoracic ratio of this population was 50%. Diabetes nephropathy (28.2%) and chronic glomerulonephritis (26.6%) were the predominant causes of ESRD and the majority of the patients (84.3%) received hemodialysis using an arteriovenous fistula. 157 patients (50.3%) had been dialyzed against a 1.0 mEq/L potassium dialysate at least once. The 1.0 mEq/L potassium dialysate users had a higher predialysis serum potassium concentration. Mean age, gender, dialysis vintage, number of patients dialyzed against a 2.5 mEq/L calcium dialysate, comorbid conditions including coronary artery disease, diabetes mellitus, preserved ejection fraction, electrocardiographic findings, and cardiothoracic ratio, cause of ESRD, type of vascular access, Kt/V, and serum albumin, hemoglobin, creatinine, phosphate, and alkaline phosphatase did not differ between the 1.0 mEq/L potassium dialysate users and nonusers.

**Table 1:** Baseline patient characteristics according to dialysate potassium concentration.

Baseline characteristics	All patients (N = 312)	1 mEq/L K_D_ Users† (N = 157)	1 mEq/L K_D_ Nonusers (N = 155)	*p* value
Age (yr)	60 ± 15	59 ± 13	60 ± 17	0.76
Female gender, n (%)	163 (52.2)	79 (50.3)	84 (54.2)	0.57
Dialysis vintage (m)	24.6 (78.2)	24.4 (77.9)	24.8 (79)	0.85
Body mass index (Kg/m^2^)	22 (4)	22 (4)	22 (4)	0.16
2.5 mEq/L Ca dialysate use, n (%)‡	66 (21.2)	35 (22.3)	31 (20)	0.72
**Comorbid condition**				
Coronary artery disease, n (%)	39 (12.5)	17 (10.8)	22 (14.2)	0.47
Diabetes mellitus, n (%)	105 (33.7)	56 (35.7)	49 (31.6)	0.52
Preserved EF, n (%)§	187/249 (75.1)	103/135 (76.3)	84/114 (73.7)	0.74
Electrocardiographic findings				
Atrial fibrillation, n (%)	16 (5.1)	11 (7)	5 (3.2)	0.21
Conduction block, n (%)	20 (6.4)	11 (7)	9 (5.8)	0.84
Atrioventricular block, n (%)	19 (6.1)	12 (7.6)	7 (4.5)	0.36
QTc prolongation, n (%)	112 (35.9)	64 (40.8)	48 (31)	0.09
Cardiothoracic ratio (%)	50 (8)	50 (9)	50 (8)	0.30
**Cause of ESRD**				0.67
Diabetic nephropathy, n (%)	88 (28.2)	45 (28.7)	43 (27.7)	
Chronic glomerulonephritis, n (%)	83 (26.6)	42 (26.8)	41 (26.5)	
Chronic interstitial nephritis, n (%)	68 (21.8)	31 (19.7)	37 (23.9)	
Hypertensive nephrosclerosis, n (%)	24 (7.7)	15 (9.6)	9 (5.8)	
Obstructive uropathy, n (%)	6 (1.9)	4 (2.5)	2 (1.3)	
Polycystic kidney disease, n (%)	5 (1.6)	3 (1.9)	2 (1.3)	
Lupus nephritis, n (%)	8 (2.6)	2 (1.3)	6 (3.9)	
Others, n (%)	30 (9.6)	15 (9.6)	15 (9.7)	
**Vascular Access**				0.92
Arteriovenous fistula, n (%)	263 (84.3)	132 (84.1)	131 (84.5)	
Arteriovenous graft, n (%)	34 (10.9)	18 (11.5)	16 (10.3)	
Cuffed hemodialysis catheters, n (%)	15 (4.8)	7 (4.5)	8 (5.2)	
**Laboratory data**				
Kt/V	1.57 ± 0.21	1.56 ± 0.19	1.57 ± 0.23	0.82
Serum albumin (g/dL)	3.97 (0.44)	3.98 (0.42)	3.94 (0.48)	0.13
Hemoglobin (g/dL)	10.5 (1.3)	10.7 (1.4)	10.5 (1.3)	0.06
Serum potassium (mEq/L)	4.7 (0.5)	4.9 (0.4)	4.6 (0.5)	<0.001
Serum creatinine (mg/dL)	9.8 ± 2.2	9.9 ± 2.1	9.8 ± 2.3	0.64
Serum phosphate (mg/dL)	4.81 ± 1.08	4.92 ± 0.93	4.7 ± 1.2	0.08
Alkaline phosphatase (U/L)	87.4 (59.3)	92.2 (60)	84.4 (58.2)	0.2

Count data are expressed as number (percentage) and continuous values are expressed as mean ± SD if normally distributed or median (interquartile range) if skewed.

Abbreviations: K_D_, potassium dialysate; Ca, calcium; EF, ejection fraction; ESRD, end-stage renal disease.

^†^Patients who had at least one prescription of a 1.0 mEq/L potassium dialysate.

^‡^Patients who had at least one prescription of a 2.5 mEq/L calcium dialysate.

^§^Preserved EF refers to patients with an ejection fraction of 55% or more.

### The effect of dialysate potassium concentration on serum potassium level

The mean predialysis serum potassium concentrations of patients during hemodialysis with 1.0 mEq/L and 2.0 mEq/L potassium dialysate were 4.8 ± 0.4 mEq/L and 4.78 ± 0.46 mEq/L, respectively (*p* = 0.46). The incidence of hypokalemia during the interval of hemodialysis with 1.0 mEq/L potassium dialysate is significantly lower than that during the interval of hemodialysis with 2.0 mEq/L potassium dialysate (6.77 and 10.21 per 100 patient-months, respectively, p<0.05).

### The influence of using 1.0 mEq/L potassium dialysate on serum potassium level

The mean predialysis serum potassium concentration when patients began to receive hemodialysis with 1.0 mEq/L potassium dialysate was 5.58 ± 0.61 mEq/L. One month after hemodialysis with 1.0 mEq/L potassium dialysate, the mean predialysis serum potassium concentration significantly decreased to a level of 4.98 ± 0.65 mEq/L (p<0.05). The incidence of hypokalemia when switching from 2.0 mEq/L to 1.0 mEq/L potassium dialysate was 5%. The mean drop of serum potassium concentrations by 1.0 mEq/L and 2.0 mEq/L in 1.0 mEq/L potassium dialysate users was 0.09 ± 0.25 mEq/L and -0.08 ± 0.18 mEq/L, respectively (p<0.05).

### Outcomes and the timing of sudden cardiac death

**Fig 2:**
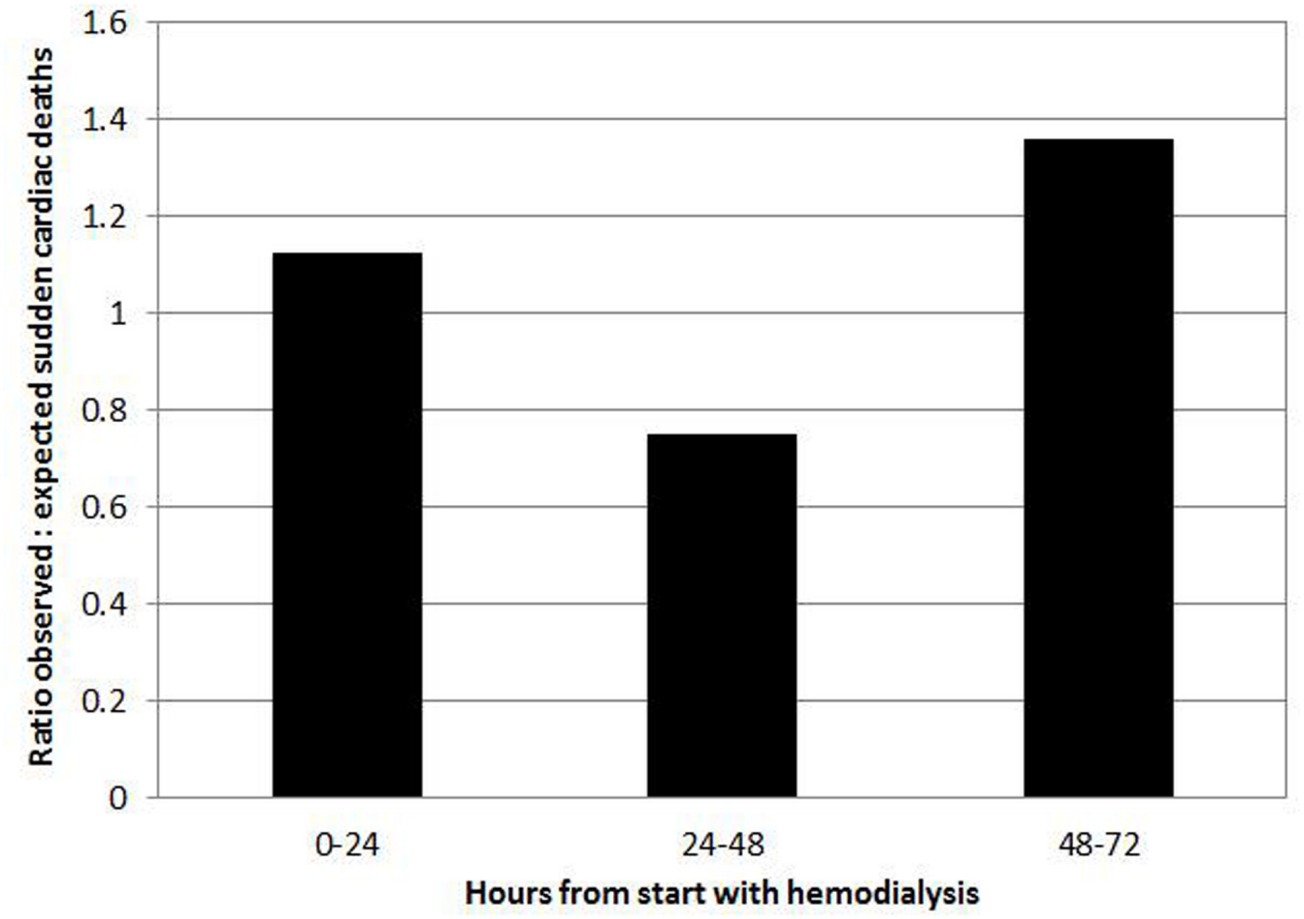
Ratio of observed to expected number of sudden cardiac death events for each 24 hour interval from the start of hemodialysis.

During the total follow-up of 1495 person-years, 16 patients (5.1%) received a kidney transplant, one (0.3%) switched to peritoneal dialysis, 47 (15.1%) had a referral to other hospital, and two (0.6%) came off hemodialysis because of sufficient renal function improvement. There were 72 deaths (23.1%), including 31 sudden cardiac deaths (9.9%) in the study. The rates of all-cause mortality and SCD were 48.17 and 20.74 per 1000 patient-years, respectively. Of the 41 non-sudden cardiac deaths, 27 were classified as being due to infection, four to malignancy, two to cerebrovascular accident, two to withdrawal of hemodialysis, two to ischemic bowel disease, two to acute myocardial infarction, one to hemorrhage, and one to heart failure. Of the 31 sudden cardiac deaths, 12 (39%) were witnessed, only one of which (3%) occurred during the hemodialysis procedure. Fig 2 depicts the ratio of observed to expected SCD proportions for the three time periods. There was a significant bimodal distribution of SCD events, with a 1.12-fold increased risk of SCD occurring in the 0–24 hour period and a 1.36-fold increased SCD risk in the 48–72 hour period (*p* = 0.017).

### Factors associated with outcomes

We used univariate and multivariate Cox proportional hazards models to evaluate the influence of predicted factors on all-cause mortality and SCD. Age, coronary artery disease, diabetes mellitus and predialysis hypokalemia with a serum potassium concentration <4.0 mEq/L had a significant adverse impact, and exposure to 1.0 mEq/L potassium dialysate, Kt/V, and serum albumin, hemoglobin, creatinine and phosphate levels had a significant protective effect on all-cause mortality in the univariate analysis (Table 2). After adjustment, age, diabetes mellitus and predialysis hyperkalemia with a serum potassium concentration >5.0 mEq/L were independent adverse predictors, and exposure to 1.0 mEq/L potassium dialysate, Kt/V, and serum albumin remained as independent protective predictors of all-cause mortality. To predict SCD, the univariate analysis revealed a significant adverse impact of age, diabetes mellitus and predialysis hypokalemia with a serum potassium concentration <4.0 mEq/L, and a significant protective effect of exposure to 1.0 mEq/L potassium dialysate, serum albumin and creatinine. However, age, diabetes mellitus and predialysis hyperkalemia with a serum potassium concentration >5.0 mEq/L were independent adverse predictors, and only exposure to 1.0 mEq/L potassium dialysate remained as an independent protective predictor in the subsequent multivariate analysis (hazard ratio = 0.33, 95% CI = 0.13–0.85). In 1.0 mEq/L potassium dialysate users, the drop of serum potassium concentrations by 1.0 mEq/L or 2.0 mEq/L did not have a significant influence on all-cause mortality (aHR, 11.8 and 0.07, respectively; 95% CI, 0.54–260.77 and 0–2.62, respectively) and SCD (aHR, 16.1 and 0.33, respectively; 95% CI, 0.21–1246.98 and 0–372.05, respectively). Furthermore, 104 participants had hyperkalemia with predialysis serum potassium concentration >5.0 mEq/L at the time of recruitment. Of these participants with baseline hyperkalemia, 72 patients had been dialyzed against a 1.0 mEq/L potassium dialysate. We included the use of 1.0 mEq/L potassium dialysate as a confounding factor and analyzed the effec of the use of 1.0 mEq/L potassium dialysate on mortality and SCD in patients with baseline hyperkalemia. In the multivariate Cox proportional hazards model, the use of 1.0 mEq/L potassium was adjusted for age, sex, dialysis vintage, body mass index, the use of 2.5 mEq/L calcium dialysate, coronary artery disease, diabetes mellitus, Kt/V, serum levels of albumin, hemoglobin, potassium, creatinine, phosphate and alkaline phosphatase. The use of 1.0 mEq/L potassium dialysate in patients with baseline hyperkalemia significantly decreased the risk of all-cause mortality (aHR = 0.02, 95% CI = 0.004–0.13) and SCD (aHR = 0.04, 95% CI = 0.004–0.34).

**Table 2:** Univariate and multivariate analyses of risk factors associated with all-cause mortality and sudden cardiac death using a Cox proportional hazards model in 312 hemodialysis patients.

	All-cause mortality		Sudden cardiac death	
	Unadjusted	Adjusted	Unadjusted	Adjusted
	HR (95% CI)	aHR (95% CI)	HR (95% CI)	aHR (95% CI)
Age	1.07 (1.05–1.09)*	1.05 (1.02–1.08)*	1.08 (1.05–1.12)*	1.07 (1.03–1.12)*
Female gender	0.83 (0.52–1.32)	1.38 (0.75–2.54)	0.93 (0.46–1.89)	0.93 (0.35–2.48)
Dialysis vintage	1 (0.99–1)	1 (1–1.006)	1 (0.99–1.01)	1.01 (1–1.02)
Body mass index	1 (0.93–1.08)	0.95 (0.87–1.03)	1.05 (0.95–1.17)	1.04 (0.9–1.2)
1.0 mEq/L K dialysate	0.17 (0.08–0.36)*	0.16 (0.07–0.37)*	0.41 (0.17–0.95)*	0.33 (0.13–0.85)*
2.5 mEq/L Ca dialysate	0.6 (0.19–1.9)	0.70 (0.21–2.3)	1.37 (0.42–4.55)	1.82 (0.5–6.6)
CAD	2.92 (1.51–5.67)*	1.89 (0.88–4.07)	2.9 (0.98–8.56)	1.7 (0.47–6.08)
DM	3.33 (2.09–5.31)*	2.09 (1.14–3.83)*	5.12 (2.47–10.6)*	4.68 (1.72–12.71)*
Kt/V	0.26 (0.08–0.85)*	0.05 (0.01–0.26)*	0.44 (0.08–2.56)	0.21 (0.01–4.4)
Serum albumin	0.07 (0.03–0.13)*	0.16 (0.06–0.4)*	0.08 (0.03–0.23)*	0.19 (0.04–1.01)
Hemoglobin	0.77 (0.62–0.97)*	0.79 (0.61–1.02)	0.99 (0.7–1.38)	0.97 (0.64–1.47)
Serum K concentration				
Serum K < 4 mEq/L	3.82 (1.5–9.7)*	1.92 (0.67–5.5)	5.37 (1.22–23.65)*	2.82 (0.52–15.4)
Serum K 4–5 mEq/L	1.00 (Reference)	1.00 (Reference)	1.00 (Reference)	1.00 (Reference)
Serum K >5 mEq/L	0.67 (0.37–1.21)	2.1 (1.08–4.09)*	1.32 (0.62–2.85)	4.11 (1.62–10.41) *
Serum creatinine	0.69 (0.61–0.79)*	0.98 (0.78–1.22)	0.66 (0.54–0.81)*	0.93 (0.65–1.34)
Serum phosphate	0.64 (0.5–0.83)*	0.91 (0.64–1.29)	0.78 (0.53–1.16)	1.14 (0.67–1.94)
ALP	1 (1–1.002)	1 (1–1.006)	1 (1–1.004)	1 (1–1.008)

Abbreviations: HR, hazard ratio; aHR, adjusted hazard ratio; CI, confidence interval; K, potassium; Ca, calcium; CAD: Coronary artery disease; DM: Diabetes mellitus; ALP: Alkaline phosphatase.

*p value < 0.05.

## Discussion

In this study, predialysis hyperkalemia defined as a serum potassium concentration >5.0 mEq/L was associated with all-cause mortality and SCD in hemodialysis patients, and the use of 1.0 mEq/L potassium dialysate in hyperkalemic hemodialysis patients with predialysis serum potassium concentrations >5.5 mEq/L significantly reduced the rate of overall mortality and SCD. The serum potassium level selected to determine the introduction of 1.0 mEq/L potassium dialysate in our unit was 5.5 mEq/L, rather than 5.0 mEq/L. Its underlying rationale was avoidance of hypokalemia, which has been implicated as a risk factor for death in hemodialysis patients [21, 22]. Our results support the findings of Kovesdy et al. who showed that the greatest survival rate of hemodialysis patients was observed when serum potassium concentrations were between 4.6 and 5.3 mEq/L, and higher potassium dialysate (>3.0 mEq/L) was associated with increased all-cause mortality in hyperkalemic hemodialysis patients with predialysis serum potassium concentrations ≥5.0 mEq/L [10]. There are several possible reasons for this association between use of 1.0 mEq/L potassium dialysate and decreased incidence of SCD. First, the present study showed a bimodal distribution of SCD events (Fig 2). There was a significant 1.36 fold increase in SCD risk in the 24 hours prior to the first hemodialysis session in a week. This finding was similar to the results of Bleyer et al., who also presented a bimodal distribution of sudden death occurrences in hemodialysis patients. They found that a threefold increase in sudden death risk occurred in the 12 hours preceding a weekly cycle, which might be attributed to hypertension or hyperkalemia during this time [17]. This suggests that the prevention of hyperkalemia in hemodialysis patients may play an important role in reducing SCD. Furthermore, we adjusted the dialysate potassium concentration according to the monthly laboratory results and the 1.0 mEq/L potassium dialysate was selectively used in hemodialysis patients with predialysis hyperkalemia. In the study by Karnik et al., who found that patients suffering from cardiac arrest were nearly twice as likely to have been dialyzed against a 0 or 1.0 mEq/L potassium dialysate, only 17.8% of patients dialyzed against a 0 or 1.0 mEq/L potassium dialysate had a predialysis serum potassium concentration ≥5.0 mEq/L [4]. Pun et al. found that the association between sudden cardiac arrest and dialysate potassium concentrations <2.0 mEq/L was significant only in hemodialysis patients with predialysis serum potassium concentrations <5.0 mEq/L [18]. Another, similar, report by Jadoul et al. showed a significant association between sudden death and dialysate potassium concentrations <3.0 mEq/L in hemodialysis patients with predialysis serum potassium concentrations <5.0 mmol/L, compared to patients with predialysis potassium concentrations >5.0 mmol/L [19]. These studies suggest that low potassium dialysate may be harmful to patients with normo- or hypokalemia, but not in patients with hyperkalemia. Adjustment of the dialysate potassium concentration to prevent hemodialysis patients with predialysis normo- or hypokalemia from being dialyzed against low potassium dialysate had been proposed [4, 18, 23, 24]. Our study demonstrates that selective use of 1.0 mEq/L potassium dialysate in hyperkalemic hemodialysis patients and adequate adjustment the dialysate composition significantly reduces SCD. Finally, some previous studies investigated peri-dialysis SCD instead of the overall SCD [4, 18]. This discrepancy may influence the interpretation of the effect of low potassium dialysate on SCD.

Rapid changes in electrolyte concentrations during hemodialysis may trigger arrhythmias. Some reports revealed that an abrupt fall in serum potassium levels by induction of dialysate potassium concentration < 3.0 mEq/L had detrimental effects on cardiac electrophysiology; either QT prolongation, ventricular ectopy or aberrant ventricular conduction [11–16]. However, it is more likely that digoxin toxicity induced by an acute decline in serum potassium concentration [11] or underlying heart diseases [12] has led to ventricular arrhythmia, rather than a direct influence of dialysate potassium level. Some reports showed that the frequency of ventricular ectopy during hemodialysis did not differ significantly in patients dialyzed with 0–2.0 mEq/L potassium dialysate [25, 26]. Moreover, using these arrhythmic surrogates for predicting SCD is questionable. A 4-year follow-up study indicated that ventricular ectopy could not predict mortality in hemodialysis patients [27]. Thus, we think that the effect of low potassium dialysate on sudden cardiac death through a possible detrimental effect of cardiac electrophysiology is still inconclusive. The impact of dialysate calcium concentrations ≤2.5 mEq/L on cardiac electrophysiology has also been reported, and was found to be more apparent when hemodialysis patients were dialyzed against a combination of low potassium and calcium [15, 16]. However, Basile et al. found that there was no significant difference in the hemodynamic stability of patients undergoing hemodialysis with dialysate calcium concentrations between 2.5 and 3.0 mEq/L [28]. The Kidney Disease Outcomes Quality Initiative (K/DOQI) clinical practice guidelines and the Kidney Disease Improving Global Outcomes (KDIGO) guidelines suggest the use of a dialysate with a calcium concentration of 2.5–3.0 mEq/L [29, 30]. In our study, patients dialyzed against a 2.5 mEq/L calcium dialysate had a dialysate potassium concentration ≥2.0 mEq/L. We did not use dialysate calcium concentrations <2.5 mEq/L. Our analysis showed that exposure to 2.5 mEq/L calcium dialysate did not increase the risk of SCD. This agrees with the results reported by Pun et al., which indicate that dialysate calcium concentrations ≥2.5 mEq/L do not increase the risk of sudden cardiac arrest [18], and is in line with the recommendations of the guidelines.

Dialysate potassium modeling to create a moderate but persistent serum-dialysate potassium gradient may reduce serum potassium levels in a gradual and effective manner during hemodialysis. Few previous studies designed to test whether dialysate potassium modeling can mitigate the arrhythmogenic effects of a fixed low potassium dialysate showed some beneficial effects on the surrogate indicators, such as reduction of premature ventricular complexes or QTc dispersion [13, 14, 31, 32]. However, randomized controlled trials are needed to test this hypothesis.

Several limitations of our study must be acknowledged. Our work is not a randomized controlled trial and we are not able to prove causality. We investigated the overall SCD rate and the causes of unwitnessed SCD were confirmed by medical records. However, we are still unable to exclude non cardiac sudden death as the cause of death completely because of the limited clinical information available. The monthly laboratory assessment for each hemodialysis patient did not include serum bicarbonate, creatine phosphokinase, and atrial natriuretic peptide. There was also lack of information about constipation and urine output. We are unable to provide these data which potentially affected serum potassium in hemodialysis patients. The exact measurement of the drop of serum potassium concentration by 1.0 mEq/L and 2.0 mEq/L potassium dialysate needed the serum potassium concentration before and immediately after dialysis using 1.0 mEq/L or 2.0 mEq/L potassium dialysate. However, the data was incomplete. Finally, this study was conducted in one center and the patients enrolled were all Asian. It may not be possible to extrapolate our results to other ethnic populations.

In conclusion, SCD is the leading cause of death in hemodialysis patients, especially in the 24-hour period starting with the hemodialysis procedure and in the 24 hours preceding a weekly cycle. Predialysis hyperkalemia increases the risk of SCD in hemodialysis patients. Using 1.0 mEq/L potassium dialysate in hyperkalemic hemodialysis patients with predialysis serum potassium concentrations >5.5 mEq/L and adequate adjustment of dialysate composition to prevent hemodialysis patients with normo- or hypokalemia from 1.0 mEq/L potassium dialysate may significantly reduce the rate of SCD.

## Supporting Information

S1 Data Underlying participant-level data is supplied in a supporting information file. (XLSX)
